# Effects of Galactomannan Oligosaccharides on Growth Performance, Mycotoxin Detoxification, Serum Biochemistry, and Hematology of Goats Fed Mycotoxins-Contaminated Diets

**DOI:** 10.3389/fvets.2022.852251

**Published:** 2022-06-24

**Authors:** Yicheng Wu, Ao Ren, Xiaokang Lv, Tao Ran, Guijie Zhang, Chuanshe Zhou, Zhiliang Tan

**Affiliations:** ^1^CAS Key Laboratory for Agro-Ecological Processes in Subtropical Region, National Engineering Laboratory for Pollution Control and Waste Utilization in Livestock and Poultry Production, Hunan Provincial Key Laboratory of Animal Nutrition Physiology and Metabolic Process, Institute of Subtropical Agriculture, Chinese Academy of Sciences, Changsha, China; ^2^University of Chinese Academy of Sciences, Beijing, China; ^3^College of Animal Science and Technology, Hunan Agricultural University, Changsha, China; ^4^College of Pastoral Science and Technology, University of Lanzhou, Lanzhou, China; ^5^School of Agriculture, Ningxia University, Yinchuan, China; ^6^Hunan Co-innovation Center of Animal Production Safety, CICAPS, Changsha, China

**Keywords:** galactomannan oligosaccharides, mycotoxins, biochemical parameters, goat, detoxification

## Abstract

This study was conducted to investigate the protective effects of mycotoxin adsorbent galactomannan oligosaccharides (GMOS) on growth performance, fermentation parameters, mycotoxins residues, serum biochemistry and oxidative stress parameters of the goats. The *in vitro* test indicated that 0.05% GMOS outperformed yeast cell wall (YCW) and montmorillonite (MMT) in aflatoxins absorption. Then 20 3-month-old *Xiangdong* black goats (15.0 ± 1.9 kg) were randomly divided into two dietary treatments for the animal test. The control group (CON group) was fed a multi-mycotoxins contaminated diet, whereas the experimental group (GMOS group) received multi-mycotoxins contaminated diet plus 0.05% GMOS. The trail lasted for 60 days, with 12 days of adaptation period and 48 days of formal experiment period. There were no treatment effects (*P* > 0.10) on growth performance, serum antioxidant capacity and activities of serum aspartate aminotransferase (AST), alanine aminotransferase (ALT), and alkaline phosphatase (ALP). The concentrations of zearalenone in the rumen were lower (*P* < 0.05) in the GMOS group. GMOS significantly reduced (*P* < 0.05) propionate concentration in the cecum, resulting in a rise (*P* < 0.01) in acetate/propionate ratio in GMOS as compared to CON. Goats of GMOS exhibited considerably greater (*P* < 0.05) levels of creatine kinase but lower (*P* = 0.02) levels of creatinine than CON. Compared with CON, GMOS supplementation significantly increased (*P* < 0.05) platelet count (PLT), platelet volume distribution width (PDW), and platelet hematocrit (PCT), while decreased (*P* < 0.05) albumin content (ALB). The 0.05% GMOS protected goats in ruminal fermentation parameters, mycotoxins residues and serum biochemistry. Moreover, GMOS had no adverse effect on goat health. To our knowledge, this is the first report of GMOS in small ruminants. These findings suggested the feasibility of dietary GMOS as a health-maintaining addictive in goat diets.

## Introduction

The prevalence of mycotoxins in feed is one of the major factors affecting feed quality, particularly in the humid and rainy areas of southern China (1). Mycotoxins can affect animal health, with poultry being the most sensitive, followed by pigs and ruminants (2). Toxins such as aflatoxins B1 (AFB1), ochratoxin A (OTA), zearalenone (ZEN) and deoxynivalenol (DON) have been detected throughout the food chain, contaminating animal products (1). Therefore, mycotoxins of feed origin are regarded as a significant threat to human and animal health worldwide. Bentonite, Zeolite and activated charcoal are widely used for mycotoxin elimination to reduce their impact (3). However, they bind to minerals and vitamins in the diet (4). As a result, researchers have concentrated their efforts on developing effective methods for removing mycotoxins from the diets without substantial nutrient loss.

Functional oligosaccharides are widely used as feed additives due to their green and non-toxic qualities (5). Both galactomannan oligosaccharides (GMOS) and mannan oligosaccharides (MOS) are functional oligosaccharides (6) and the use of GMOS and MOS as feed additives have been approved in China (7). Previous report has shown that MOS can dramatically promote the proliferation of beneficial bacteria in the gastrointestinal tract (GIT), thereby enhancing animal immune function, and regulating animal growth and metabolism (8). For ruminants, MOS maintained the overall health and performance of crossbred calves (9) and dairy calves (10). In addition, the capacity of MOS to adsorb mycotoxins such as ZEN and OTA had been well established using static *in vitro* models (11, 12).

Due to the special GIT structure of ruminants, it is commonly considered that rumen microbes could degrade oligosaccharides and weaken their activity (13). However, based on the positive effects of MOS on growth performance in sheep and MOS is an effective organic adsorbent to bind various mycotoxins while posing no toxicity to essential dietary components (6). We hypothesized that adding GMOS to goat diets would be beneficial to the adsorption of mycotoxins in the GIT, hence alleviating the deleterious impact of mycotoxins on animal health. To our knowledge, this is the first report of GMOS on the efficiency of toxin decontamination in small ruminants.

Therefore, the current study sought to evaluate the effects of GMOS supplementation in mycotoxin-contaminated diets on the growth and health of goats and establish a theoretical basis for the use of GMOS as a mycotoxin adsorbent in goat feeds.

## Materials and Methods

The present study included an *in vitro* mycotoxin adsorption experiment and a goat feeding trial. The *in vitro* experiment was carried out at the Institute of Subtropical Agriculture, Chinese Academy of Sciences, Changsha, China in May 2019; while the animal study was conducted at the *Xiangdong* Black Goat Reproduction Center, Liuyang, Hunan province, China from 29 April 2019 to 27 June 2019.

### *In vitro* Toxins Adsorption Test

The adsorbents used in the *in vitro* adsorption test included GMOS, yeast cell wall (YCW) and montmorillonite (MMT). The GMOS was kindly provided by Jiangsu Kangwei Biologic Co., Ltd. (Jiangsu, China); yeast cell wall (YCW) and montmorillonite (MMT), provided by Xuzhou Saifu Biological Co., Ltd. (Xuzhou, China), were used as controls to determine the toxin adsorption efficiencies of GMOS. All other reagents were of analytical grade or higher and purchased from Jiangsu Kangwei Biologic Co., Ltd. (Jiangsu, China). Feed materials were stored at 37°C and 15% humidity for a week to let them mildew (Supplementary Figure 1). Feed samples were collected, dried, pooled, and ground through a 1 mm screen for AFB1 extraction. Five grams of feed powder was weighed into a 50 mL conical tube (Thermo Fisher Scientific, Waltham, USA) that contained 20 mL of a mixture of methanol: water (1:1 v/v). The mixture was vigorously mixed on a multitube vortexer for 10 min, followed by centrifugation at 4,000 rpm for 5 min. The supernatants were collected and filtered through 0.45 μm filters (Thermo Fisher Scientific, Waltham, USA), used as testing samples. Adsorbent solutions of different concentrations (0.00, 0.01, 0.02, 0.03, 0.04, 0.05, 0.06, 0.07, 0.08, and 0.09%) were prepared by diluting GMOS with ultra-pure water. The *in vitro* adsorption experiment was then performed as described by Saleemi et al. (12). Then the concentration of GMOS with the best toxin adsorption efficiency was selected for further comparision with conventional adsorbents such as YCW (0.05%), MMT (0.05%) following the methods described by Marroquin et al. (14). The concentrations of AFB1 extracted from feed samples treated with or without adsorbents were measured using an enzyme-linked immunosorbent assays (ELISA) kit (Neogen Co., Shanghai, China) according to the description of Aazami et al. (15).

### Animal Management and Dietary Treatments

Twenty *Xiangdong* goats (3-month-old, 15.0 ± 0.1 kg) were randomly divided into two feeding treatments. The control group (CON) was fed a naturally contaminated diet, while the GMOS group (GMOS) was fed the control diet supplemented with 0.05% GMOS, which was added to the naturally contaminated diet in the mixer as the last step in mixing. The ingredients and nutrient compositions, and the initial mycotoxin concentrations of the basal experimental diets are illustrated in Table 1.

**Table 1:** Ingredients and nutrient compositions of the experimental diet (DM basis).

**Ingredients**	**Contents (%)**	**Nutritional level**	**Contents (%)**
Corn stalk	50.00	ME (MJ/kg)^1^	2.52
Corn	24.44	CP (%)	12.69
Soybean meal	8.00	EE (%)	35.06
Soy protein concentrate	6.45	NDF (%)	31.00
Fat powder	8.09	ADF (%)	21.00
Salt	0.60	RDP (%)	6.91
Premix^2^	1.00	RUP (%)	5.78
Calcium carbonate	0.70	Calcium (%)	0.21
Calcium hydrogen phosphate	0.72	Phosphorus (%)	0.16

*ME, metabolic energy; CP, crude protein; EE, ether extract; NDF, neutral detergent fiber; ADF, acid detergent fiber; RDP, rumen digested protein; RUP, rumen undigested protein.*

*^1^ME was calculated according to Zhang (16).*

*^2^Premix composition (Premix/kg): provided per kilogram of diet: vitamin A, 419,491.5254 IU/kg; vitamin E, 12,711.86441 IU/kg; Ferric, 1.5 g/kg; Copper, 0.5 g/kg; Cobalt, 0.0055 g/kg; Iodine, 0.0075 g/kg.; Manganese, 3 g/kg; Zinc, 2.5 g/kg; Selenium, 0.0025 g/kg*.

The animal study lasted 60 days, with 12 days of pre-feeding and 48 days of formal experiment. All goats were housed individually in stainless steel metabolic cages (150 × 60 × 80 cm) with plastic slatted floors. Goats were individually fed a naturally contaminated diet twice daily (08:00 and 18:00) with the same amount of diet. All goats had free access to clean water. During the feeding trial, body weight was recorded weekly before morning feeding. Feed offered and refusals for each goat were recorded daily throughout the experiment period to calculate the dry matter intake (DMI), average daily gain (ADG), and feed to gain ratio (F/G).

### Sample Collection and Handling

Refusals and experimental diets were sampled for chemical analysis. Feed samples were oven-dried at 65°C for 72 h, ground to pass through a 1-mm sieve and sealed in bags before chemical analysis. Blood samples were obtained by jugular venipuncture before morning feeding on the 27^th^ and 28^th^ of the formal experimental period. A pair of 5 ml blood samples were collected into test tubes (Guangzhou Improve Medical, China), among which one contained EDTA for the routine hematological parameters and the other tube contained no anticoagulants for the serum biochemical analysis. After 30 min still standing, samples were centrifuged at 3,500 rpm for 15 min, 1 ml plasma was taken for antioxidant capacity and biochemical parameters analysis. At the end of the experiment, all goats were fasted for 24 h and water was withheld for 12 h. The animals were weighed, electrically stunned, and exsanguinated under commercial procedures. One hundred milliliter rumen content and 20 g cecum content were extracted upon euthanasia with an alcohol-cleaned spatula and stored at −80°C. Cecal digesta was diluted with deionized water. After vortexing, ~10 ml rumen content and cecum content were centrifuged at 10,000 rpm for 15 min at 4°C, 1 ml supernatant was transferred into tubes containing 0.1 ml of 25% (w/v) metaphosphoric acid, and then frozen at −20°C for subsequent determination of volatile fatty acids (VFAs) and ammonia nitrogen (NH_3_-N) concentration. Frozen feed materials, ruminal and cecal content samples were thawed, dried, pooled, and ground through a 1 mm screen for AFB1, ZEN and DON extraction.

### Samples Analyses

Feed samples and feces were used for nutrient composition analysis. The dry matter (DM) (method 934.01), ash (method 927.02), ether extract (EE, method 920.39), crude protein (CP, method 976.06), calcium (Ca, method 978.02), total phosphorus (TP, method 946.06) were determined according to AOAC (17). Neutral detergent fiber (NDF) and acid detergent fiber (ADF) contents were determined using a Fibretherm Fiber Analyzer with F57 filter bags (ANKOM A200, ANKOM Technology Corp., Fairport, NY, USA) according to Van Soest et al. (18). Rumen degradable protein (RDP), rumen undegradable protein (RUP) were calculated according to the description of Kekana et al. (19). The AFB1, ZEN and DON residues in the ruminal and cecal contents of the two treatments and the basal diet were carried out according to the description of Guo et al. (20).

Volatile fatty acids and NH_3_-N concentrations were measured according to Chen et al. (21). Hematological examination of blood samples was performed following the standard method reported by Bafti and Mozaffari (22) using a CELL-DYN 3700 analyzer. Routine hematological parameters include white blood cell (WBC), mononuclear cells (MON), eosinophil granulocyte counts (EOS), basophil granulocyte counts (BAS), neutrophils (NEU), lymphocyte (LYM), red blood cell counts (RBC), platelet count (PLT), platelet volume distribution width (PDW), and platelet hematocrit (PCT) were tested. The detection of serum antioxidant capacity, including total antioxidant capacity (T-AOC), superoxide dismutase (SOD) and malondialdehyde (MDA) levels were performed using commercial kits according to the manufacturer's protocols (Nanjing Jiancheng Bioengineering Institute, Nanjing, China). Serum activities of alanine aminotransferase (ALT), aspartate aminotransferase (AST), alkaline phosphatase (ALP) and creatine kinase (CK) and concentrations of albumin (ALB), blood urea nitrogen (BUN) and creatinine (CREA) were determined using an automatic biochemistry analyzer (Sekisui Medical Co. Ltd., Tokyo, Japan) using commercially available kits (Nanjing Jiancheng Bioengineering Institute, Nanjing, China).

### Statistical Analysis

All data were processed preliminarily by Excel. Then the data of the growth performance, toxins concentration, fermentation parameters, serum biochemistry, and hematology indexes of the two groups were analyzed using the Shapiro–Wilk test for normal distribution and followed by student *t*-test to compare means using SPSS 19.0 (SPSS Inc., Chicago, USA) (23). Statistical significance was defined as *P* < 0.05, and tendencies at 0.05 ≤ *P* < 0.10.

## Results

### Ability of GMOS to Adsorb Mycotoxins *in vitro*

**Figure 1:**
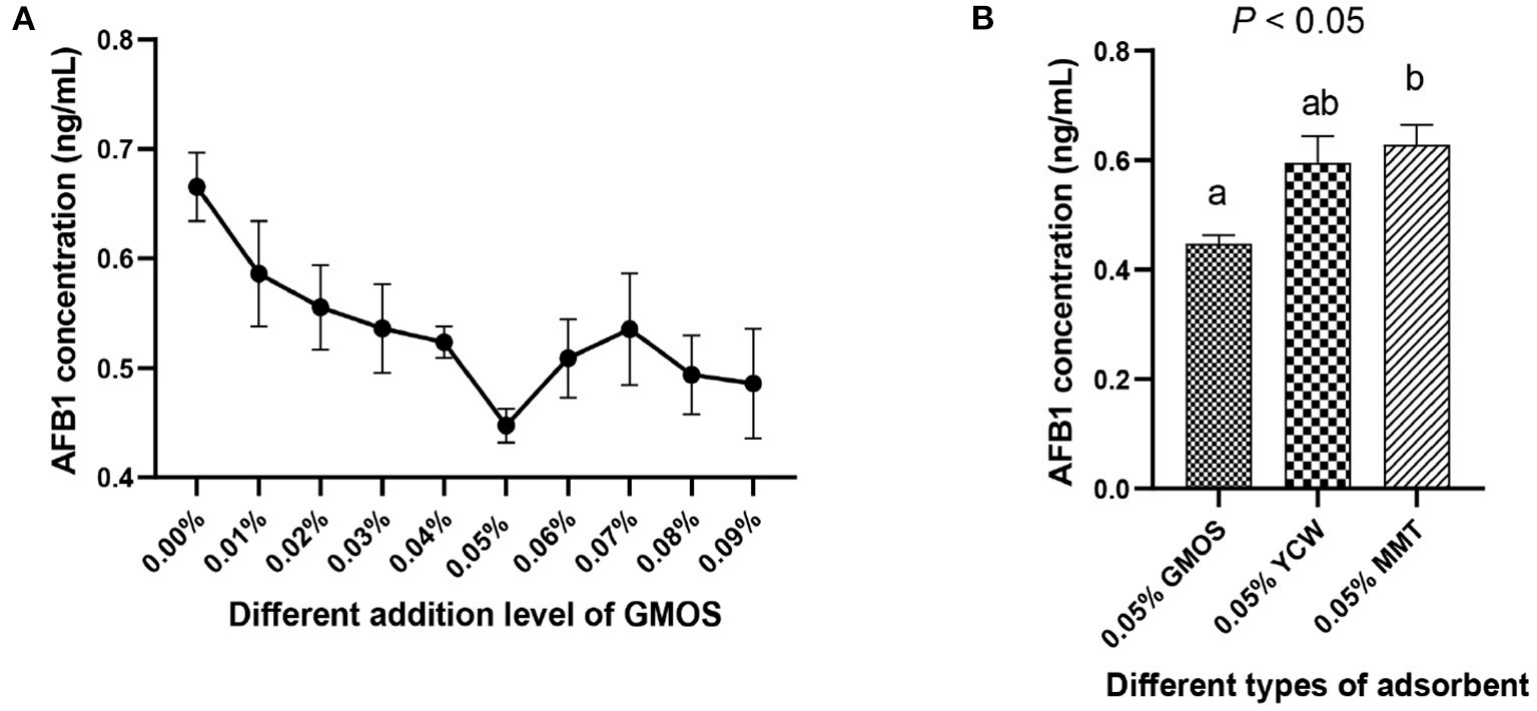
The ability of GMOS to adsorb mycotoxins (Aflatoxins B1, AFB1), which were measured *in vitro* using ELISA. **(A)** Effects of different addition levels of GMOS (0.00, 0.01, 0.02, 0.03, 0.04, 0.05, 0.06, 0.07, 0.08, and 0.09%) on AFB1 concentration. **(B)** Effects of 0.05% GMOS, 0.05% Yeast cell wall (YCM), 0.05% Montesserstone (MMT) on AFB1 concentration. ^a, b^ Mean values within a row with different superscript letters were significantly different (*P* < 0.05).

The results of GMOS on adsorbing aflatoxins in a naturally contaminated diet are presented in Figure 1. The added level of 0.05% GMOS showed the lowest AFB1 concentration (Figure 1A), suggesting the best efficacy in adsorbing AFB1. Furthermore, the concentration of AFB1 in 0.05% GMOS treat was significantly lower (*P* < 0.05) than 0.05% YCW and 0.05% MMT treats (Figure 1B), indicating that 0.05% GMOS outperformed traditional adsorbents like YCW and MMT in absorption of aflatoxins.

### Dry Matter Intake, Average Daily Gain, and Feed to Gain Ratio

The DMI, ADG and F/G of goats from GMOS treatment were numerically more remarkable than that of CON goats in Table 2; however, not reach statistically significant differences (*P* > 0.10).

**Table 2:** Effects of dietary supplementation of GMOS on growth performance in goats.

**Parameter**	**Treatments** ^**1**^		**SEM**	***P*-Value**
	**CON**	**GMOS**		
Initial BW (kg)	9.91	10.10	0.34	0.79
Final BW (kg)	11.66	12.02	0.41	0.69
DMI (g/d)	332.52	347.45	10.29	0.49
ADG (g/d)	35.57	42.62	3.38	0.32
F/G	11.43	12.06	0.79	0.71

*BW, body weight; DMI, dry matter intake; ADG, average daily gain; F/G, feed to gain ratio; SEM, standard error of the mean.*

*^1^CON, control diet, without additive; GMOS, control diet + 0.05% GMOS*.

### Mycotoxins Concentration in the Gastrointestinal Tracts

The concentrations of mycotoxins including AFB1 and DON did not differ between CON and GMOS treatments either in ruminal or cecal digesta; whereas, the concentration of ZEN in rumen was significantly lower (*P* < 0.01) in GMOS treatment than that in CON (Table 3). Notably, the concentration of ZEN in cecal digesta was greater than that in ruminal digesta.

**Table 3:** Effects of dietary supplementation of GMOS on concentrations of toxins in the gastrointestinal digesta in goats.

**Gastrointestinal**	**Parameters**	**Treatments** ^**1**^		**SEM**	***P*-Value**
		**CON**	**GMOS**		
Rumen	AFB1 (μg/kg)	5.33	5.09	0.17	0.48
	ZEN (μg/kg)	42.08^a^	35.40^b^	1.32	0.01
	DON (mg/kg)	0.27	0.29	0.028	0.77
Cecum	AFB1 (μg/kg)	4.04	3.82	0.19	0.57
	ZEN (μg/kg)	104.13	100.07	7.78	0.80
	DON (mg/kg)	0.23	0.20	0.018	0.43

*^a, b^Mean values within a row with different superscript letters were significantly different (P < 0.05).*

*AFB1, aflatoxins B1; ZEN, zearalenone; DON, deoxynivalenol; SEM, standard error of the mean.*

*^1^CON, control diet, without additive; GMOS, control diet + 0.05% GMOS*.

### Fermentation Parameters

The results of NH_3_-N concentration, total and individual VFA, and A/P ratio was presented in Table 4. The amounts of NH_3_-N, TVFA, and the molar proportions of butyric acid, isobutyric acid, valeric acid and isovaleric acid in the rumen or cecum did not differ (*P* > 0.10) between CON and GMOS treatments. In the rumen, the molar proportion of propionic acid was greater (*P* < 0.05) in GMOS than that in CON, and the molar proportion of acetic acid tended to be lower (*P* = 0.08) in GMOS than that in CON, thus leading to a tendency of lower (*P* = 0.07) A/P ratio in GMOS. On the contrary, the molar proportion of acetic acid in the cecum did not differ between CON and GMOS treatments, but the molar proportion of propionic acid was lower (*P* < 0.05) in GMOS than in CON, resulting in a more significant (*P* < 0.01) A/P ratio in GMOS.

**Table 4:** Effects of dietary supplementation of GMOS on ruminal and cecal fermentation parameters in goats.

**Gastrointestinal**	**Parameters**	**Treatments** ^**1**^		**SEM**	***P*-Value**
		**CON**	**GMOS**		
Rumen	TVFA (mmol/L)	51.09	46.74	2.04	0.26
	Acetate (A) (%)	69.31	66.64	0.80	0.08
	Propionate (P) (%)	22.67^b^	25.32^a^	0.72	0.05
	Butyrate (%)	3.01	2.82	0.68	0.79
	Iso-butyrate (%)	1.93	1.75	0.14	0.82
	Valerate (%)	1.05	1.20	0.05	0.18
	Iso-valerate (%)	2.68	2.87	0.13	0.48
	Proportion A:P	3.06	2.69	0.11	0.07
	NH_3_-N (mg/100 ml)	17.50	15.07	1.19	0.45
Cecum	TVFA (μmol/g)	48.95	52.09	3.08	0.63
	Acetate (A) (%)	70.15	70.86	0.96	0.72
	Propionate (P) (%)	14.95^a^	13.60^b^	0.36	0.03
	Butyrate (%)	10.74	9.14	0.63	0.18
	Iso-butyrate (%)	1.28	1.14	0.04	0.13
	Valerate (%)	1.48	1.34	0.07	0.33
	Iso-valerate (%)	1.10	0.98	0.04	0.13
	Proportion A:P	4.72^b^	5.45^a^	0.14	<0.01
	NH_3_-N(mg/g)	0.16	0.14	0.01	0.32

*^a, b^Mean values within a row with different superscript letters were significantly different (P < 0.05).*

*^1^CON, control diet, without additive; GMOS, control diet + 0.05% GMOS.*

*SEM, standard error of the mean; TVFA, total volatile fatty acid*.

### Hematology, Serum Biochemical and Enzyme Parameters

The serum antioxidant indexes including SOD, T-AOC and MDA were not (*P* > 0.10) different between CON and GMOS treatments (Table 5). Table 6 shows the impact of GMOS supplementation on hematologic parameters and serum biochemical indexes of goats. Compared to CON, the percentage of MON, PDW and PCT and counting of PLT were greater (*P* < 0.05) in the GMOS supplemented group; whereas, the other indexes did not differ between the two treatments. As for the biochemical parameters, the Albumin (ALB) and creatinine (CREA) levels were significantly lower (*P* < 0.05) in the GMOS group compared to the CON group. In contrast, the serum creatine kinase (CK) concentrations were markedly higher (*P* < 0.05) in goats fed GMOS than those fed only a control diet.

**Table 5:** Effects of dietary supplementation of GMOS on serum antioxidant indexes of goats.

**Parameter**	**Treatments** ^**1**^		**SEM**	***P*-Value**
	**CON**	**GMOS**		
SOD (U/ml)	34.741	37.14	1.73	0.51
T-AOC (U/ml)	1.20	1.18	0.01	0.13
MDA (nmol/L)	2.95	2.89	0.22	0.83

*SOD, superoxide dismutase; T-AOC, total anti-oxidation competence; MDA, malonaldehyde; SEM, standard error of the mean.*

*^1^CON, control diet, without additive; GMOS, control diet + 0.05% GMOS*.

**Table 6:** Effect of dietary supplementation of GMOS on hematological and serum biochemical parameters in goats.

**Item**	**Treatments** ^**1**^		**SEM**	***P*-Value**
	**CON**	**GMOS**		
**Hematological parameters**				
WBC (10^9^/L)	14.47	14.32	0.27	0.75
MON (%)	2.86^b^	4.06^a^	0.22	0.01
EOS (%)	5.24	4.90	0.67	0.81
BAS (%)	1.53	1.70	0.09	0.36
NEU (%)	44.24	26.79	1.99	0.16
LYM (%)	45.88	50.84	2.24	0.29
RBC (10^12^/L)	15.64	14.16	0.43	0.08
PLT (10^9^/L)	93.56^b^	224.00^a^	29.74	0.03
PDW (%)	13.38^b^	13.69^a^	0.07	0.01
PCT (%)	0.029^b^	0.074^a^	0.01	0.03
**Biochemical parameters**				
ALB (g/L)	34.22^a^	32.53^b^	0.29	0.01
ALT (U/L)	25.62	26.91	0.96	0.52
AST (U/L)	103.33	114.52	3.52	0.13
ALP (U/L)	3.66	3.08	0.35	0.33
CK (U/L)	259.12^b^	296.38^a^	8.07	0.02
BUN (mmol/L)	6.30	6.33	0.09	0.78
CREA (μmol/L)	45.12^a^	41.28^b^	0.66	0.01

*^a, b^Mean values within a row with different superscript letters were significantly different (P < 0.05).*

*WBC, white blood cell; MON(%), mononuclear cells percentage; EOS, eosinophil granulocyte counts; BAS, basophil granulocyte counts; NEU, neutrophils; LYM, lymphocyte; RBC, red blood cell counts; PLT, platelet count; PDW, platelet volume distribution width; PCT, platelet hematocrit; ALB, albumin; ALT, alanine amino transferase; AST, aspartate amino transferase; ALP, alkaline phosphatase; CK, creatine kinase; BUN, blood urea nitrogen; CREA, creatinine; SEM, standard error of the mean.*

*^1^CON, control diet, without additive; GMOS, control diet + 0.05% GMOS*.

## Discussion

In the current study, the *in vitro* test results indicated that 0.05% GMOS outperformed traditional adsorbents like YCW and MMT in the absorption of aflatoxins. However, there was no difference in DMI, F/G, or ADG between the GMOS and the CON treatments. Previous studies have reported that dietary supplementation of MOS has been proven to enhance the performance of monogastric animals, including BW gain and feed conversion efficiencies (5, 24, 25). However, investigations on MOS in ruminants have yielded inconsistent results. Hill et al. (26) observed no differences in DMI and ADG when 3 g/day of a MOS was added to the diet of young Jersey calves. Similarly, Westland et al. (27) reported that dietary supplementation of 2 g of MOS to each calf daily did not improve the performance of calves. However, some other studies demonstrated enhanced ADG, AFDI, and feed efficiency in calves when a greater amount of MOS (4 g/calf/day) was supplemented in the diet (9) or milk replacer (10). Therefore, the growth performance of goats was not well improved as expected in the present study, likely attributed to the low dose of GMOS.

The *in vivo* test revealed that toxin concentrations in the GIT were much lower than those detected in the diet (refer to Suppplementary Table 1). These were likely due to two reasons: on the one hand, the toxins were adsorbed by mannan products (24); on the other hand, the complex GIT bacteria can degrade mycotoxins to different degrees (28). Although GMOS effectively adsorbed AFB1 in *in vitro* test, the concentrations of AFB1 did not differ between CON and GMOS treatments either in ruminal or cecal digesta, suggesting the importance of carrying out *in vivo* animal study. DON and AFB1 are metabolized into less toxic substances by rumen microorganisms (29), while ZEN is commonly transferred to metabolites like α-zearalenol (α-ZEL) and β-zearalenol (β-ZEL), which are significantly more hazardous than ZEN (30). Currently, it is challenging to separate ZEN and its metabolites using ELISA assay due to their similar structures. Moreover, rumen microbes can also selectively utilize oligosaccharides as fermentation substrates (31). Fewer GMOS reached the cecum, which is insufficient to change the mycotoxin concentration in the cecum. Therefore, the greater concentration of ZEN in cecal digesta than that in ruminal digesta was reasonable. Better analysis methods are needed to determine ZEN and its metabolites in the future. Our results suggested that GMOS could decrease the concentration of ZEN in the rumen; however, an insufficient amount of GMOS could adsorb ZEN in the cecum.

Then we investigated how GMOS affects the animal's fermentation parameters. GMOS did not change the TVAF concentration and the ruminal ammonia concentration. It significantly enhanced the molar proportion of propionate in the rumen but decreased that in the cecum. Wang et al. (31) have reported that mycotoxins negatively impact ruminal and intestinal fermentation. In contrast, MOS can attenuate the adverse effects of mycotoxins by promoting the growth of beneficial bacteria such as *Lactobacillus* in the rumen, and *Lactobacillus* is essential for the production of butyric and propionic acids (8). So we speculate that GMOS could also repair the detrimental effects of mycotoxins by promoting the growth of beneficial rumen bacteria. Furthermore, the small amount of GMOS reached the cecum, and the accumulation of toxins, especially ZEN, inhibited the growth of beneficial bacteria in the cecum.

Mycotoxins cause liver lesions and kidney damage with subsequent changes of some enzymatic parameters in goats (20). Previous studies have shown that MOS have antioxidative properties in sheep and chickens, including increasing the serum levels of SOD and T-AOC while lowing serum MDA levels (32, 33). In the current study, dietary supplementation GMOS had no effect on antioxidant indices in goats, including SOD, T-AOC and MDA. This finding suggested that GMOS supplementation would not cause any oxidative stress to small ruminants. Unaffected serum EOS and BAS levels used as stress indicators could also be robust support (34), suggesting that GMOS supplementation did not cause stress to goats. Similarly, dietary supplementation MOS did not affect the WBC count, EOS or BAS of dairy cows (35). Zheng et al. (36) reported that MOS did not significantly influence basal hematological parameters because their effects on the animal body are slight. In the current study, dietary supplementation of GMOS significantly increased routine blood indicators like MON, PCT, PLT and PDW. Previous study has shown a decrease in PLT and PDW (37), MON and PCT (8) in pigs exposed to mycotoxins such as ZEN and DON. Generally, an increase in MON indicates enhanced resistance to pathogens (38). In addition, platelet-related parameters such as PCT, PLT and PDW are associated with inflammatory reactions and immune responses (39). The rising level of PCT, PLT and PDW indicated the potential of GMOS to affect hematological parameters. We speculate that GMOS could prevent mycotoxin attacks on the goats' immune system to some extent.

In general, mycotoxins reduces the concentrations of CK and the decrease in serum levels of this substance may reflect renal cell damage with leakage of the contents into the blood (40). The current experiment showed an elevated CK level in the GMOS group, suggesting that GMOS may mitigate the damage caused by mycotoxins to the kidneys. Nargeskhani et al. (41) indicated that animals challenged by mycotoxins showed lower ALB level, the dropping level of serum ALB content indicated liver damage in animals. Although the current experiment showed decreased ALB content in the GMOS group, the ALB level was within the normal range (42). This is the first time we have found similar results. Furthermore, the serum levels of ALT, AST, and ALP were widely used to evaluate liver functions (41). The serum levels of ALT, AST, and ALP showed no differences between two treatments. Our results suggested that the addition of GMOS did not impair the serum biochemistry of goats. The similar result showed that the addition of mannan in diets had no deleterious effects on serum biochemistry and hematological characteristics of laying hen (43). These facts suggested that addition GMOS to goats' diet had no negative effects on their liver function. The presence of an increase in serum CREA level indicates the damage of kidney function (44). In our study, CREA level was significantly decreased (*P* < 0.05) in the GMOS group, suggesting that feeding a naturally contaminated diet to goats might impair kidney function and supplementation of GMOS could mitigate the nephrotoxicity. The decreased CREA level observed in our study was similar to the result reported by Raja et al. (45).

## Conclusion

It was concluded that GMOS at 0.05% level was beneficial in promoting the proliferation of beneficial rumen bacteria. GMOS could also alleviate mycotoxin injury on the liver and kidney. Further research is needed to evaluate the degradation of mycotoxins by rumen microorganisms. And more *in vivo* experiments should be conducted to adjust the amount of GMOS in the diet for the adsorption effect of various mycotoxins. However, we can affirm the ability of GMOS as a green mycotoxin adsorbent. GMOS could serve as an economical solution to mycotoxicosis and make livestock feeding more cost-effective.

## Data Availability Statement

The original contributions presented in the study are included in the article/Supplementary Material, further inquiries can be directed to the corresponding authors.

## Ethics Statement

The animal study was reviewed and approved by Animal Care and Use Guidelines of the Animal Care and the Use Committee, Institute of Subtropical Agriculture, Chinese Academy of Sciences, Changsha, PR China (Approval Number: ISA-2019-0115).

## Author Contributions

YW, AR, CZ, and ZT designed the experiment. YW, AR, and XL conducted the experiment. YW, AR, XL, and GZ collected and analyzed data. YW prepared the tables, prepared the figures, and wrote the manuscript. CZ acquired funding. TR and CZ revised the manuscript. All authors contributed to the article and approved the submitted version.

## Funding

This work was jointly supported by the National Natural Science Foundation of China (U20A2057 and 31730092) and National Basic Research and Development Program of China (2018YFD0501903).

## Conflict of Interest

The authors declare that the research was conducted in the absence of any commercial or financial relationships that could be construed as a potential conflict of interest.

## Publisher's Note

All claims expressed in this article are solely those of the authors and do not necessarily represent those of their affiliated organizations, or those of the publisher, the editors and the reviewers. Any product that may be evaluated in this article, or claim that may be made by its manufacturer, is not guaranteed or endorsed by the publisher.
