# Supplementary material for: Effects of Galactomannan Oligosaccharides on Growth Performance, Mycotoxin Detoxification, Serum Biochemistry, and Hematology of Goats Fed Mycotoxins-Contaminated Diets
Source: Front Vet Sci. 2022 Jun 24;9:852251. doi: 10.3389/fvets.2022.852251 (PMC9263622; doi:10.3389/fvets.2022.852251)
Supplement: Supplementary file 1 [file Table_1.DOCX]

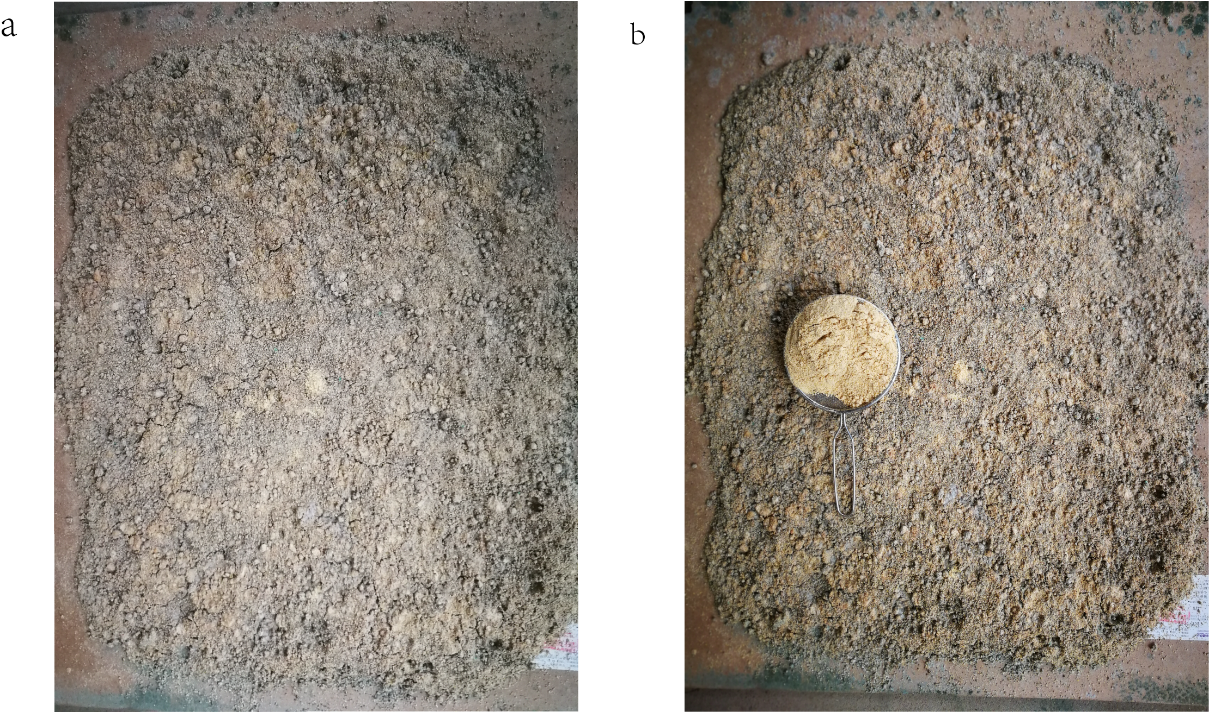


**Supplementary figure 1** a. Natural mycotoxins-contaminated diet; b. Comparison of normal diet to mycotoxins-contaminated diet.

**Supplementary table 1** Mycotoxin concentrations of the naturally contaminated diet (DM basis).

| **Items**^1^ | **Concentration** |
| --- | --- |
| AFB1 (µg/kg) | 30 |
| ZEN (µg/kg) | 350 |
| DON (mg/kg) | 2 |

^1^ AFB1, aflatoxins B1; ZEN, zearalenone; DON, deoxynivalenol.
